# The Efficacy and Tolerability of the Clonidine Transdermal Patch in the Treatment for Children with Tic Disorders: A Prospective, Open, Single-Group, Self-Controlled Study

**DOI:** 10.3389/fneur.2017.00032

**Published:** 2017-02-23

**Authors:** Pan-Pan Song, Li Jiang, Xiu-juan Li, Si-Qi Hong, Shuang-Zi Li, Yue Hu

**Affiliations:** ^1^Department of Neurology, Children’s Hospital Affiliated to Chongqing University of Medical Sciences, Chongqing, China; ^2^Ministry of Education Key Laboratory of Child Development and Disorders, Chongqing, China; ^3^Key Laboratory of Pediatrics in Chongqing, CSTC2009CA5002, Chongqing, China; ^4^Chongqing International Science and Technology Cooperation Center for Child Development and Disorders, Chongqing, China

**Keywords:** clonidine transdermal patch, tic disorders, children, prediction model, efficacy

## Abstract

**Background:**

To evaluate the efficacy and tolerability of a clonidine transdermal patch in the treatment of children with tic disorders (TD) and to establish a predictive model for patients.

**Methods:**

Forty-one patients who met the inclusion criteria entered into 12 weeks of prospective, open, single-group, self-controlled treatment with a clonidine transdermal patch. The Yale Global Tic Severity Scale (YGTSS) was employed before therapy (baseline) and at 4, 8, and 12 weeks after therapy.

**Results:**

(1) The total effect rates of treatment with a clonidine transdermal patch were 29.27, 53.66, and 63.41% at 4, 8, and 12 weeks, respectively. Compared with the baseline, the differences were significant at three different observation periods. (2) Compared to the level of 25% reduction, there were significant decreases in the score-reducing rate of motor tic and total tic severities at 12 weeks. (3) If the disease course was ≤24 months and the motor tic score was <16 at the baseline, there was an effective rate of 100% for treatment with the clonidine transdermal patch. If the disease course was ≤24 months and the motor tic score was >16, there was an effective rate of 57.1%. If the disease course was >24 months and the clinical classification was chronic TD, there was an effective rate of 62.5%. If the disease course was >24 months and the clinical classification was Tourette’s syndrome, 90% of the patients were invalid. (4) The main adverse events were rash, slight dizziness, and headache.

**Conclusion:**

(1) When patients were pretreated with a D2-dopamine receptor antagonist that was ineffective or not tolerated well, switching to a clonidine transdermal patch treatment was effective and safe. (2) A clonidine transdermal patch could be a first-line medication for mild and moderate TD cases that are characterized by motor tics.

## Introduction

Tic disorders (TD) commonly occur among youth and are classified into three types: TD, chronic motor or chronic vocal/phonic tics (CTD), and Tourette’s syndrome (TS). The prevalence of these types is 10–15, 3–4, and 1%, respectively (1). All TD are more common in boys than in girls (male/female ratio of approximately 4:1), and the clinical course may be transient or chronic. Typically, tic onset occurs in early childhood (i.e., 4–12 years), usually at the age of 7–8 years (2, 3). It peaks in severity and prevalence in preadolescence (i.e., 10–12 years) and begins to decline during adolescence. Approximately, one-half to two-thirds of adolescents with TS have a decrease in tic severity by early adulthood (4, 5). It is characterized by the presence of sudden, rapid, non-rhythmically repetitive and mainly involuntary body movements (motor tics) and/or vocalizations (phonic tics). TD ranges in location (e.g., face and legs) and complexity (e.g., eye blinks, facial grimacing, jaw, shoulder shrugging, multistep movements, and phrases). Most TS patients also suffer from comorbid conditions such as attention deficit hyperactivity disorder (ADHD), obsessive-compulsive disorder, or depression.

At present, the pathogenesis of TD remains unclear. Some scholars have suggested that D2-dopamine receptor dysfunction in the basal ganglia may be the major cause of tics. The principal treatment is pharmacological treatment combined with psychological behavior therapy, and individualization is emphasized (6). Many affected children or adolescents do not require pharmacological treatment because their tics do not impair social and academic functioning. When persistent tics impact the quality of life, social and academic functioning and lifetime activities and psychological behavior therapy fails, pharmacological treatment (e.g., D2-dopamine receptor antagonists, selective monoamine antagonists, alpha-2-adrenoreceptor agonists, and other drugs) should be considered (1, 7–10). D2-dopamine receptor antagonists, such as haloperidol and tiapride, are often effective but poorly tolerated, so their use is limited (1, 2, 11).

Recently, dysregulation of the noradrenoreceptor system has been suggested as the underlying pathophysiology. Exacerbation due to stress is a characteristic of tics (12). Clonidine, which acts as an alpha-2-adrenoreceptor agonist, has been used to treat hypertensive patients. Small doses of clonidine can alleviate tics by activating presynaptic autoreceptors in the locus ceruleus and then reducing norepinephrine release and turnover. Since the early 1980s, it has been reported that clonidine has beneficial effects in the treatment of TD (13–15), especially for patients with ADHD (1, 16).

A clonidine transdermal patch is a transdermo-therapeutic system that releases drugs at a constant speed successively for 7 days. Compared with the ordinary preparation, a clonidine transdermal patch has no peak or valley plasma concentration, so it can achieve full efficacy and reduce adverse effects. Changing in weekly intervals only vastly improves compliance in children.

We analyzed the clinical data and follow-up results of 41 patients who were pretreated for more than 2 months with D2-dopamine receptor antagonists using a prospective, open, single-group, self-controlled study. This study was undertaken with multiple objectives: (1) to evaluate the efficacy and tolerability of a clonidine transdermal patch in the treatment of TD and (2) to establish a prediction model for subjects that can provide evidence-based recommendations for appropriately choosing clonidine transdermal patches for treatment.

## Subjects and Methods

### Subjects

The inclusive criteria were as follows: (1) between the ages of 6 and 18 years, weight more than 20 kg, normal intelligence and either sex; (2) met the diagnostic criteria of the Diagnostic and Statistical Manual of Mental Disorders, fourth edition (DSM-IV; American Psychiatric Association, 1994) for TD (17); (3) received pretreatment for more than 2 months with dopamine receptor antagonists but had a Yale Global Tic Severity Scale (YGTSS) score-reducing rate of ≤25% (18) or was not well tolerated; (4) written informed consent and assent were obtained from patients who were more than 7 years old and from their parents.

Subjects were terminated from participation in the study if they met one or more of the following criteria: (1) in violation of the study protocol during the study period; (2) lost to follow-up during the study period; (3) unused clonidine transdermal patches for more than 2 weeks; (4) occurrence of any serious adverse events.

This study was approved by the ethics committee of the Children’s Hospital of Chongqing Medical University and was conducted in accordance with the latest version of the Declaration of Helsinki.

### Study Design and Measurements

All subjects had a comprehensive and detailed assessment of their current health status, which included pulse rate, blood pressure, respiratory rate, height, and weight, before treatment and at 4, 8, and 12 weeks after therapy. Every subject completed the examination of routine hematology, routine urinalysis, liver and renal functions, fasting blood glucose, and electrocardiogram before therapy. All TD medications were discontinued before therapy for 1 week, and subjects entered the therapy group after completing the washout period. Subjects were prohibited from using alpha-2-adrenoreceptor blockers, antidepressants or other psychoactive medications, psychological behavior therapy, and transcranial magnetic stimulation during the study period. Subjects who did not want to continue to participate in the study because of a poor therapeutic effect were considered invalid. To prevent bias in the statistical results, we used the last observation carried forward (LOCF) to analyze the data so that we did not exclude these patients from the study.

The tic symptoms of each participant were evaluated by an appointed professional using the YGTSS (19) at baseline (after completing the washout period and before therapy) and at weeks 4, 8, and 12.

The Total Tic Score (TTS) = the motor tic score + the phonic tic scores (range = 0–50).

The Global Severity Score (TGSS) = the motor tic score + the phonic tic score + overall impairment rating (range = 0–100).

Severity grading was divided into mild, moderate, and severe, and the TGSSs ranged from 0–25, 25–50, and 50–100, respectively.

### Drug and Dosing Schedule

Clonidine patches were provided by Shanxi RFL Pharmaceutical Co., Ltd. The drug specifications were as follows: 1.0, 1.5, and 2.0 mg sizes. When the weights of the patients were >20 and ≤40, >40 and ≤60, and >60 kg, the doses of the drug were 1.0, 1.5, and 2.0 mg per week, respectively. The clonidine transdermal patch was placed on the back under the shoulder blades, and the two sides were alternated weekly. The old patch was not removed until the new one had been placed for 1 day.

### Evaluation

Using the YGISS score that was measured after completion of the washout period before the therapy as the baseline score, we compared the score-reducing rate of four factors at three separate time points, including baseline and the end of weeks 4, 8, and 12. The contents of the evaluation included the score-reducing rate for motor tics, phonic tics, the total tic score, and the overall impairment rating. The definitions were as follows

(1) Score-reducing rate for Motor Tic Score = (Motor Tic Score at baseline − Motor Tic Score at a different period)/Motor Tic Score at baseline × 100%;
(2) Score-reducing rate for Phonic Tic Score = (Phonic Tic Score at baseline − Phonic Tic Score at a different period)/Phonic Tic Score at baseline × 100%;
(3) Score-reducing rate for Total Tic Score = (Total Tic Score at baseline − Total Tic Score at a different period)/Total Tic Score at baseline × 100%;
(4) Score-reducing rate for Overall Impairment Rating = (Overall Impairment Rating at baseline − Overall Impairment Rating at a different period)/Overall Impairment Rating at baseline × 100%.

The definition of clinical efficiency was a YGTSS score-reducing rate of >25% reduction. We took the score-reducing rate of the total tic score as the judgment standard for a curative effect. Clinical recovery was defined as the score-reducing rate of the total tic score >75%; obvious improvement as 50% < score-reducing rate ≤75%; improvement as 25% < score-reducing rate ≤50%; and clinical invalidation as score-reducing rate ≤25%. Clinical efficiency = clinical recovery + obvious improvement + improvement.

### Data Analyses

Statistical analysis was performed using SPSS22.0 statistical software package. The quantitative data were evaluated by paired *t*-tests, a one-sample *t*-test, and analysis of variance. The categorical data were evaluated by a chi-squared test and Fisher’s exact probability test. We conducted the efficacy prediction for our data by building a decision tree. The LOCF was used as the end point for a participant. Statistical significance was established at *p* < 0.05.

## Results

A total of 41 subjects were enrolled (34 males, 7 females; male/female ratio 4.8:1; age range 6–16 years, average 9.21 ± 2.07 years, median 9 years).

Clinical classifications were TD in 7 patients (7/41, 17.0%), CTD in 17 patients (17/41, 41.5%), and TS in 17 patients (17/41, 41.5%).

The disease severity grading was moderate in 28 patients (28/41, 68.3%) and severe in 13 patients (13/41, 31.7%).

The course of the disease ranged from 2 to 109 months; the average was 27.36 ± 21.70 months, and the median was 24 months. All subjects had a history of using D2-dopamine receptor antagonists (e.g., tiapride, haloperidol + artane, and tiapride + topamax), the course of which ranged from 2 months to 5+ years. Of the 41 patients, 2 dropped out of the treatment at 4 weeks, and 7 dropped out at 8 weeks due to inefficacy. One patient dropped out at 8 weeks for clinical control of tics.

### Curative Effect Analysis

At 4 weeks, clinical recovery occurred in 1 patient (1/41, 2.44%), obvious improvement occurred in 1 patient (1/41, 2.44%), improvement occurred in 10 patients (10/41, 24.39%), and invalidation occurred in 29 patients (29/41, 70.73%) (Table 1). The total efficacy rate was 29.27% (12/41). A significant difference (*p* < 0.001) was observed compared with baseline.

**Table 1:** **Changes in efficacy for the clonidine transdermal patch at different treatment stages**.

Different stages	Effectiveness				
	Clinical recovery (%)	Obvious improvement (%)	Improvement (%)	Invalidation (%)	Total rate of effectiveness (%)
4 weeks	2.44	2.44	24.39	70.73	29.27***
8 weeks	7.32	34.15	12.20	46.34	53.66***^,▲^
12 weeks	36.59	14.63	12.20	36.59	63.41***^,▲▲^

Compared with baseline, ***p < 0.001; compared with 4 weeks, ^▲^p < 0.05, ^▲▲^p < 0.01; compared with 8 weeks.

At 8 weeks, clinical recovery occurred in 3 patients (3/41, 7.32%), obvious improvement occurred in 14 patients (14/41, 34.15%), improvement occurred in 5 patients (5/41, 12.20%), and invalidation occurred in 19 patients (19/41, 46.34%). The total efficacy rate was 53.66% (22/41). A significant difference was observed between baseline (*p* < 0.001) and 4 weeks (χ^2^ = 5.025, *p* < 0.05).

At 12 weeks, clinical recovery occurred in 15 patients (15/41, 36.59%), obvious improvement occurred in 6 patients (6/41, 14.63%), improvement occurred in 5 patients (5/41, 12.20%), and invalidation occurred in 15 patients (15/41, 36.59%). The total efficacy rate was 63.41% (26/41). A significant difference was observed between baseline (*p* < 0.001) and 4 weeks (χ^2^ = 9.612, *p* < 0.01). There was no significant difference between the 8 and 12 week values (χ^2^ = 0.804, *p* > 0.05). This finding suggested that the onset time of the clonidine transdermal patch should be 4 weeks, and the peak time was 8–12 weeks.

### All Factors of YGTSS Score-Reducing Rates at Different Periods

The definition of clinic efficiency was a YGTSS score-reducing rate for each factor that was >25% reduction. Compared with the level of 25% reduction, we conducted a one-sample *t*-test (*p* < 0.05) and found a significant difference in the score-reducing rate of motor tics and total tics at 12 weeks, whereas there was no significant difference in the score-reducing rate of phonic tics and the overall impairment rating at different stages (Table 2). Compared with 4 weeks, there was a significant difference in the score-reducing rate for motor tics and total tics at 8 weeks and in each factor at 12 weeks (*p* < 0.05). Compared with 8 weeks, there was no significant difference in the score-reducing rate for each factor at 12 weeks (*p* > 0.05). For intraobserver comparisons, there was some improvement in phonic tics and overall impairment ratings at 12 weeks, but these scores did not reach the criteria of clinical efficacy (25% reduction). It is suggested that a clonidine transdermal patch could improve motor tics but has poor results for phonic tics and overall impairment ratings.

**Table 2:** **Changes in YGTSS score-reducing rate (%) at different treatment stages**.

YGTSS score-reducing rate	Different stages				
	4 weeks (mean ± SD)	8 weeks (mean ± SD)	12 weeks (mean ± SD)	*F*	*p*
Motor tics	16.74 ± 24.75	35.55 ± 34.61^▲^	46.65 ± 36.55**^,▲▲▲^	8.717	0.000
Phonic tics	−8.50 ± 51.69	16.68 ± 51.13	24.47 ± 57.76^▲^	2.453	0.094
Total tic	8.52 ± 33.94	32.21 ± 38.19^▲^	45.11 ± 41.29**^,▲▲▲^	9.816	0.000
Overall impairment rating	12.19 ± 34.31	25.40 ± 37.82	37.60 ± 44.77^▲▲^	4.308	0.016

Clinical effectiveness for treatment was more than 25% in YGTSS score-reducing rate; compared with the lever of 25%, derived from one-sample t-test, **less than 0.01; compared with 4 weeks, ^▲^p < 0.05, ^▲▲^p < 0.01, ^▲▲▲^p < 0.001; compared with 8 weeks.

*YGTSS, the Yale Global Tic Severity Scale*.

### Forecast Analysis

**Figure 1:**
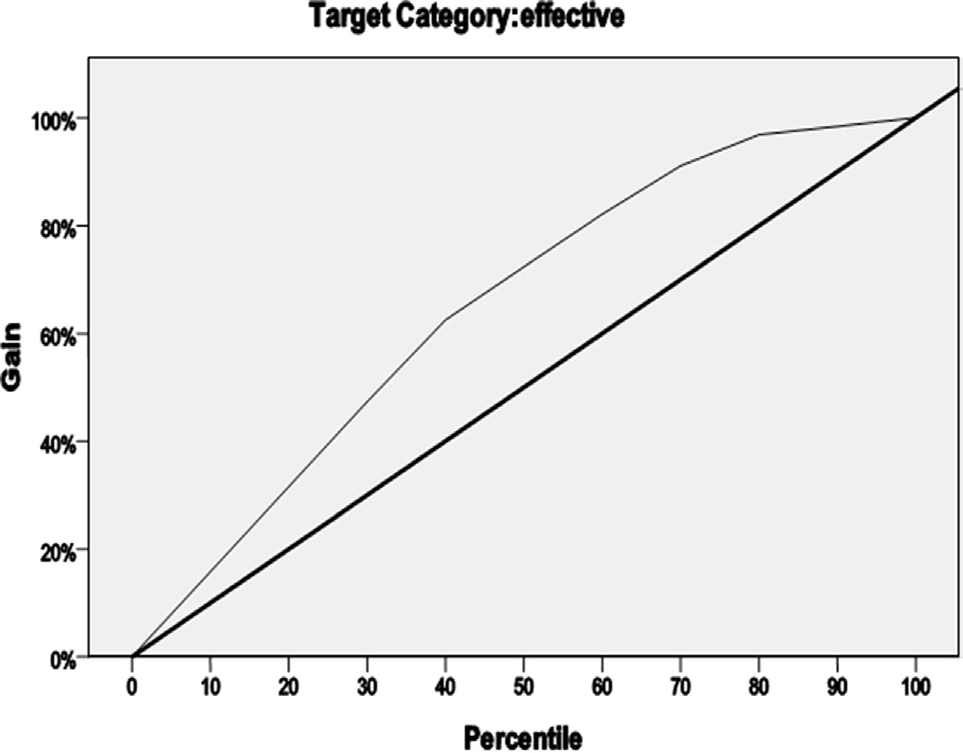
**Growing method of classification and regression tree**.

We defined treatment outcomes depending on whether they were effective (YGTSS score-reducing rate for each factor for >25% reduction) as a dichotomous dependent variable. There were 11 independent variables (e.g., gender, onset age, age at first treatment, the age and weight at enrollment, the duration of the illness, clinical classification, comorbid ADHD, motor tic score at baseline, phonic tic score at baseline, and overall impairment rating at baseline). On that basis, we designed a four-armed decision tree to predict the conditions for effective treatment with a clonidine transdermal patch. The model used a classification and regression tree as the growing method and used the method of cross-validation to avoid excessive fitting. The rightness rate of the model was 82.9%, which indicates high predictive accuracy and reliability. The gain is shown in Figure 1.

**Figure 2:**
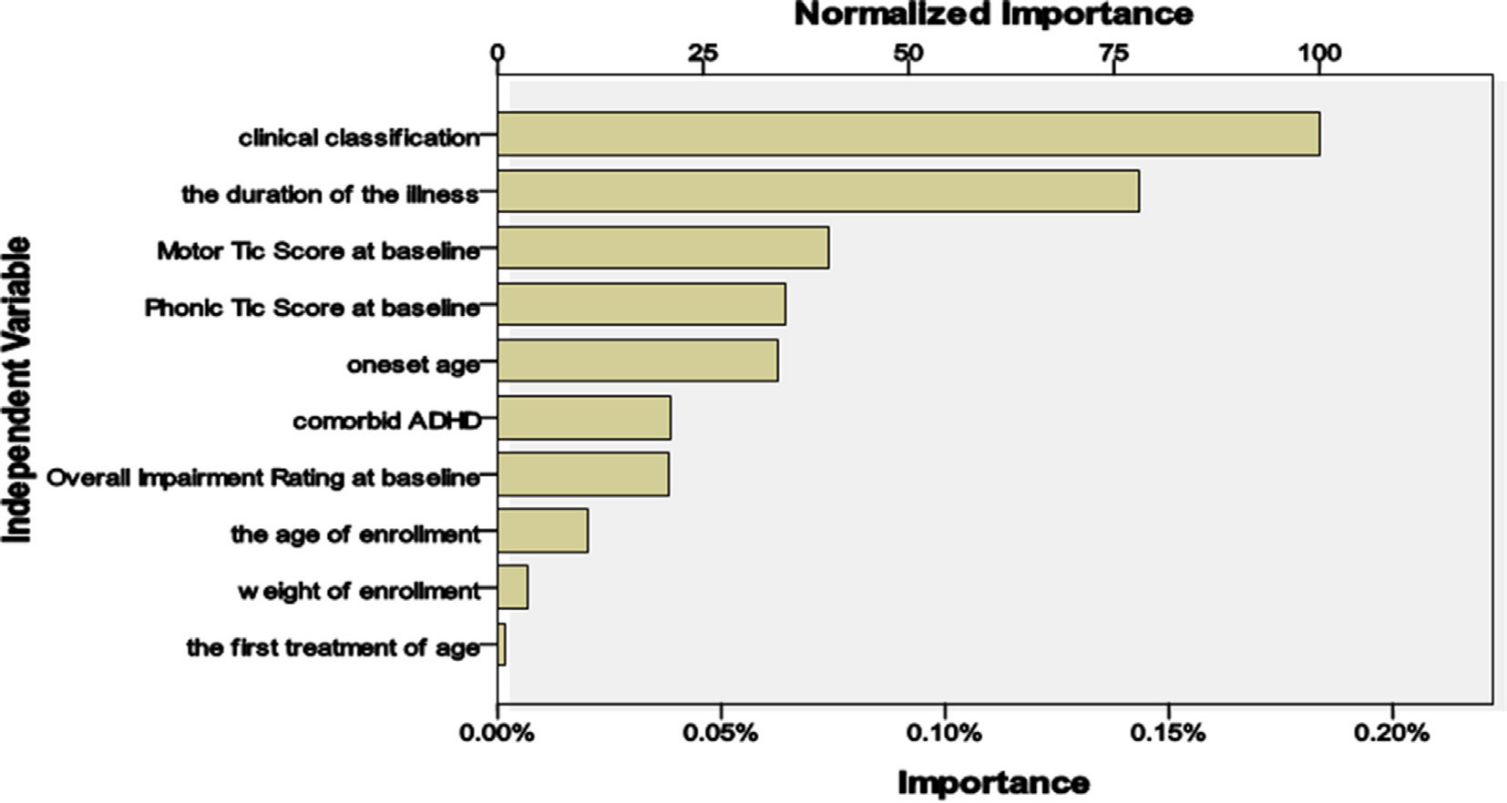
**The importance of independent variables affecting treatment**.

The model showed the following: (1) there was a treatment efficacy of 100% with the clonidine transdermal patch if the disease course was ≤24 months and the motor tic score was <16; (2) there was an efficacy of only 57.1% if the disease course was ≤24 months and the motor tic score was >16; (3) there was an efficacy of 62.5% if the disease course was >24 months and the clinical classification was CTD; and (4) 90% of patients who were treated with a clonidine transdermal patch were invalid if the disease course was >24 months and the clinical classification was TS. The importance of the independent variables in relation to treatment is summarized in Figure 2. The most important variables were classification (100%), course of the disease (78.1%), and motor tics (40.4%).

### Safety and Tolerability Evaluation

During the 12-week test period, adverse events occurred in 4.76% (2/41) of the subjects, but no subject withdrew from the study due to adverse events. One child reported a rash where the clonidine transdermal patch was placed, and another had a hematuria at week 9. The hematuria may have been unrelated to the clonidine transdermal patch because it disappeared before the drug was withdrawn.

As of June 2015, we had successfully followed up with 37 patients. A total of 56.7% (21/37) of the patients willingly chose a clonidine transdermal patch after the 12-week test ended, the courses of which lasted between 4 and 17 months.

A total of 38.1% (8/21) of patients who were treated with a clonidine transdermal patch obtained complete remission of tics and were relapse free. Although 4.8% (1/21) of the patients achieved complete remission of tics, they recrudesced after 6 months. Our follow-up results showed long-term tolerability for patients with a clonidine transdermal patch.

There were eight cases that had adverse events during follow-up after the end of the clinical trial. The most common adverse events were rash (33.3%, 7/21), slight dizziness, and headache (9.5%, 2/21, one combined with rash), which were mild in severity and reasonably well tolerated. There were no severe adverse events with regard to changes in systolic or diastolic blood pressure or pulse.

## Discussion

The YGTSS is the most widely used measure for treatment responses for tics. The YGTSS is based on the severity of motor and phonic tics in five separate dimensions: number, frequency, intensity, complexity, and interference. Each of these dimensions is scored on a 0–5 scale according to tic severity. The total tic score (range 0–50), which consists of the symptoms of tics, is derived by adding the total motor tic Score (range 0–25) and the total phonic tic score (range 0–25). The YGTSS also includes a separate impairment rating focused on distress and impairment experienced in interpersonal, academic, and occupational realms, the summation of which is the so-called overall impairment rating (range 0–50). A global severity score (range 0–100) is derived by summing the total motor tic score, the total phonic tic score, and the overall impairment rating. Because the overall impairment rating often remained unchanged over one week, the total tic score is more suitable for assessment of tic symptom severity. Jeon et al. (18) verified that a 25% reduction in the total tic score provided optimal sensitivity (87%) and specificity (84%) for predicting positive responses. Thus, we defined clinical efficacy as a YGTSS score-reducing rate of >25% reduction. We took the score-reducing rate of the total tic score as a judgment standard for a curative effect.

A dysregulation of neurotransmission and abnormal receptors is believed to be the underlying pathophysiology of tics. It is widely acknowledged that dopamine receptor hypersensitivity in the basal ganglia is the pathogenesis of TD. The dopamine D2 receptor antagonist has achieved the most reliable and fastest treatment response compared to other medications and is therefore the mainstay of any pharmacological treatment. Haloperidol, which is a typical dopamine antagonist and is most commonly used, may improve tic symptoms in 70–80% of patients (8, 20) and has an A level of evidence (21). However, this agent may be associated with more and more serious side effects than other neuroleptics. The significant adverse effects, especially extrapyramidal symptoms such as acute dystonia or akathisia, sedation, weight gain, and decline in cognitive functions, have led to its discontinuation. Tiapride, although associated with low side effects, is less effective than haloperidol and has a B level of evidence (21). Many studies have suggested that alpha-2 agonists, such as clonidine, are less effective than antipsychotics or atypical neuroleptics, such as haloperidol, aripiprazole, and risperidone (10, 22, 23). We found that the effective rates of treatment with a clonidine transdermal patch were 29.27, 53.66, and 63.41% at weeks 4, 8, and 12, respectively. Compared with the baseline, significant changes were observed at three different observation periods. Because our subjects were unaffected by or not tolerant to dopamine receptor antagonists, it can be speculated that switching to a clonidine transdermal patch may be effective if dopamine antagonists are ineffective or intolerable. The study of Du et al. (24) showed that clonidine improved tic symptoms in 68.31% of patients after 4 weeks of treatment and showed a significant change compared with placebo. Zhong et al. (25) reported that there were no significant differences in efficacy between clonidine and tiapride. In our studies, compared with week 4 of treatment with a clonidine transdermal patch, significant changes in the effective rate were observed at weeks 8 (*p* < 0.05) and 12 (*p* < 0.01). However, there were no significant differences between weeks 8 and 12 (*p* > 0.05). It was concluded that the onset time of clonidine may be after the first 4 weeks, and the peak time may be at 8–12 weeks. It has been suggested that clonidine needs to be taken for a longer period (3 weeks or more) than haloperidol to lead to improvement (26, 27). Thus, withdrawal should not be undertaken because of ineffectiveness within a short time. It has been reported that medications for treatment of TD are helpful initially and have a noticeable effect but leave tics unchanged or worsened after approximately a year or 6 months (28). Our long-term follow-up of 8–17 months showed that 38.1% (8/21) of patients who were treated with a clonidine transdermal patch achieved complete remission of tics and were relapse free. Thus, clonidine had definite efficacy in the long term.

Further analysis of all factors of YGTSS score-reducing rates at different periods showed that compared with the level of 25% reduction, there were no significant differences in the score-reducing rate of four factors, including motor tics, phonic tics, total tics, and overall impairment, at 4 and 8 weeks. Compared with the level of 25%, there were significant differences in the score-reducing rate of motor tics and total tics at 12 weeks. This finding suggested that a clonidine transdermal patch could improve motor tics but had poor efficacy for phonic tics and overall impairment ratings. This finding is similar to the results of studies by Leckman et al. (29, 30) and Du et al. (24). Clonidine is more effective for motor tics than for phonic tics, which may be because motor tics are less influenced by psychological factors.

The conditional fluctuation of tics was frequent, and individual differences were remarkable. Treatment protocols should be individualized and analyzed by synthesis according to treatment history, course of the disease, classification, comorbidities, and age (16). Our model showed that the first three important independent variables affecting treatment were classification (100%), course of the disease (78.1%), and motor tics (40.4%). Hanna (31) suggested that dopamine antagonists should be used in treating severe cases and (or) when other pharmaceutical treatments failed to improve the tics. Clonidine can be used as the first-line treatment for mild to moderate cases, especially for children and patients receiving initial treatment. Other studies (32–34) have suggested that clonidine can also be used as refractory and (or) in severe cases or cases with co-occurring ADHD. Our forecast analysis showed that there was an efficacy of 100% for treatment with a clonidine transdermal patch if the clinical course was ≤24 months and motor tic score was <16. The efficacy was only 57.1% if the clinical course was ≤24 months and the motor tic score was >16. There was an efficacy of 62.5% if the clinical course was >24 months and clinical classification was CTD. However, 90% of patients who were treated with a clonidine transdermal patch were invalid if the clinical course was >24 months and clinical classification was TS. This finding provides the basis for the conditions in which clonidine transdermal patch treatment should be chosen. However, there was a limitation in the predictive value of the model because of the small sample size.

Haloperidol is often poorly tolerated in TD, with only 20–30% of patients continuing to take this medication over time (35) secondary to the serious side effects. Clonidine, however, often has good tolerance (13–15, 22, 24, 26, 30, 36). The most common adverse events with clonidine are sedation and dizziness, which often tend to be mild to moderate in severity (16, 26) without evidence of other adverse effects, including cardiac toxicity (16). In our study, the most common adverse events were rash (33.3%, 7/21), slight dizziness and headache (9.5%, 2/21); no patients dropped out of treatment due to adverse effects. In our long-term follow-up of 8–17 months, 56.7% (21/37) of patients continued to take this medication after the 12-week test. This result implies that clonidine has good tolerance and a high level of safety over time.

Our study was an open-label trial that was not placebo controlled. It could be argued that the findings were the result of a placebo effect rather than reflecting the intrinsic activity of the study drugs. However, compared with other diseases with a psychological origin, TD intrinsically has a lower placebo effect (37, 38). The duration of illness for the enrolled children ranged from 2 months to several years or longer, and there were no good responses to D2-dopamine receptor antagonists. Furthermore, placebo effects often do not last long. In our study, clonidine continued to improve the symptoms of TD for the 4–12 weeks of the study period and maintained its effects in the long-term follow-up.

In conclusion, when patients were pretreated with a D2-dopamine receptor antagonist that was ineffective or not tolerated well, switching to a clonidine transdermal patch treatment was effective and safe. A clonidine transdermal patch could be a first-line medication for mild and moderate TD cases that are characterized by motor tics.

## Ethics Statement

We confirm that we have read the Journal’s position on issues involved in ethical publication and affirm that this report is consistent with those guidelines.

## Author Contributions

P-PS conducted the acquisition and interpretation of the data and was involved in drafting the manuscript; YH conceived the study, participated in its design, and helped to draft the manuscript; LJ critically revised the study for important intellectual content; X-jL, S-QH, and S-ZL participated in the design of the study and performed the statistical analysis. All authors declare that they have no competing interests. All authors read and approved the final manuscript.

## Conflict of Interest Statement

The authors declare that the research was conducted in the absence of any commercial or financial relationships that could be construed as a potential conflict of interest.
